# Patient and Therapist Expectations for a Blended Cognitive Behavioral Therapy Program for Depression: Qualitative Exploratory Study

**DOI:** 10.2196/36806

**Published:** 2022-12-30

**Authors:** Ece Atik, Magnus Schückes, Jennifer Apolinário-Hagen

**Affiliations:** 1 Ruhr University Bochum Bochum Germany; 2 Institute for SME Research and Entrepreneurship University of Mannheim Mannheim Germany; 3 Institute of Occupational, Social and Environmental Medicine, Faculty of Medicine, Centre for Health and Society Heinrich Heine University Dusseldorf Dusseldorf Germany

**Keywords:** blended cognitive behavioral therapy, bCBT, cognitive behavioral therapy, digital health, mental health, internet, mobile app, blended psychotherapy, depression, user perspectives, mobile phone

## Abstract

**Background:**

Blended cognitive behavioral therapy (bCBT)—the combination of digital elements and face-to-face psychotherapy—has been proposed to alleviate challenges that patients and therapists face in conventional cognitive behavioral therapy. There is growing evidence that adding digital elements to face-to-face psychotherapy can contribute to better treatment outcomes. However, bCBT programs still show considerable shortcomings, and knowledge on how to improve digital apps using a bCBT protocol is limited.

**Objective:**

This study aimed to inductively identify functions and qualities that are expected from a bCBT treatment for depression in the eyes of patients and psychotherapists who were not currently receiving or practicing bCBT treatment.

**Methods:**

We used a qualitative exploratory study design and conducted 3 focus group interviews (n=6 in each) and 5 semistructured in-depth interviews with therapists as well as 11 individual interviews with patients with a primary diagnosis of depression and currently undergoing cognitive behavioral therapy treatment in Germany. Themes and categories were established inductively from transcribed interview records based on a rigorous coding method.

**Results:**

Both therapists and patients expected a digital app to provide patients with the opportunity to track their mood, work on therapeutic homework activities, easily access an intervention set for harder moments, and efficiently facilitate administrative tasks. The desire to be able to customize bCBT protocols to individual patient circumstances was evident in both patient and therapist interviews. Patients differed with respect to what content and the amount of material the app should focus on as well as the method of recording experiences. Therapists viewed digital apps as potentially aiding in their documentation work outside of sessions. Different attitudes surfaced on the topic of data security, with patients not as concerned as therapists.

**Conclusions:**

Both patients and therapists had substantially positive attitudes toward the option of an integrated bCBT treatment. Our study presents novel findings on the expectations and attitudes of patients and therapists.

## Introduction

### Background

Depression is one of the most common mental health–related diagnoses worldwide [1]. It causes debilitating psychological pain, problems in one’s social life, and loss of productivity to individuals who have it. Furthermore, the hopelessness that many patients with depression feel is linked to suicidal behavior [2]. For many individuals, depression has a recurring trajectory over the life span, and each recurrent episode increases the likelihood of development of later episodes [3]. Beyond the hardships that individuals go through, depression also imposes a considerable burden on health care systems worldwide as the psychological and pharmaceutical treatments for depression are costly [4].

Cognitive behavioral therapy (CBT) is an effective and well-researched psychotherapy approach with extensive evidence on its effectiveness in the treatment of a wide range of mental health disorders, including major depression and dysthymia [5-7]. Especially for the treatment of depression, CBT has been shown to provide superior treatment outcomes compared with alternative psychotherapy methods [8]. In CBT, a high degree of adherence to the therapy is key to a successful treatment process and long-term outcomes. Insufficient engagement with therapeutic resources (especially between sessions) and dropouts from psychotherapy because of poor engagement are potential hazards for patients that may impair its effectiveness [9]. Research shows that patients’ adherence to assigned homework within their treatment program—especially behaviorally demanding assignments—is considerably low [10,11]. According to therapists, more than half of patients face difficulties in completing their homework as prescribed, which in some cases leads to considerable negative consequences for therapy outcomes [12]. In contrast, there is a wealth of evidence that homework compliance is a significant predictor of better treatment outcomes [13-16]. Given these circumstances, it is not surprising that relapse and recurrence rates are high in CBT treatments. For example, a meta-analysis addressing relapse and recurrence rates following CBT programs for depression reported that relapse and recurrence rates can be as high as 46.5% throughout the treatment and highly predicted by residual depressive symptoms after the completion of therapy [17].

To alleviate these problems and support patients in CBT adherence, scholars have proposed to leverage the advantages of internet-based CBT (iCBT) in conventional face-to-face CBT treatment plans [18]. iCBT treatment options consist of web-based platforms offered to patients for use through smartphones or computers that provide them with psychoeducational content and homework tasks digitally [19]. By integrating an iCBT app and face-to-face CBT treatment into the same treatment protocol, a blended CBT (bCBT) modality offers a new treatment method that aims to incorporate the merits of both [20]. Although it promotes patient autonomy and makes psychotherapy easily accessible outside the therapy sessions, bCBT still preserves personal contact with a therapist and recognizes the therapeutic relationship as an important factor in treatment success [21]. bCBT programs have been suggested to intensify the therapy process by making it easier to record a patient’s activities and thoughts, review the content of the therapy between sessions, and organize homework. Organizing homework in particular leads patients to focus on completion and enhances learning as a result [22]. bCBT programs also promise to reduce the total therapy duration [23,24].

Although there is no consensus in the literature on a single taxonomy for bCBT programs [18,25,26], bCBT treatments have primarily been categorized as either *sequential* or *simultaneous* according to how the face-to-face and digital components of blended therapy are introduced and scheduled [18]. In sequential bCBT treatments, patients’ individual work with a digital mental health app either precedes or follows the face-to-face treatment with a psychotherapist [18]. Stepped care approaches could exemplify sequential blended treatments [27,28]. Simultaneous bCBT programs offer face-to-face and digital elements within the same period [18], oftentimes in the form of working on therapeutic homework through the digital app between face-to-face sessions with a therapist [29,30]. Our focus in this study is on simultaneous bCBT treatment as we believe this approach has the greatest potential to efficiently achieve positive long-term therapy goals.

Several studies have confirmed the additional benefits of simultaneous bCBT compared with face-to-face therapy in alleviating depression symptoms and mental health–related burdens on patients [18,25,29,31-33], whereas there are also studies where patient mental health outcomes in bCBT conditions were found to be similar to usual treatment [21,34-36]. Important is that none of those studies found that bCBT resulted in worse mental health outcomes. Similarly, some studies find bCBT to be a more cost-effective alternative to standard CBT [24,34,37], whereas others find longer total treatment time and higher costs in bCBT conditions when the amount of therapist contact remains constant [21,38]. More research is needed to shed light on these various outcome findings. The bCBT programs in these studies face shortcomings as they are rarely developed as a unique synthesis of the digital app and face-to-face therapy but simply use them simultaneously as distinct or *adjunct* therapy elements [29,39]. As an alternative to adjunct programs, more personalized and interconnected digital and face-to-face elements would offer an *integrated* bCBT program [25,30].

### Objectives

To overcome the obstacles associated with conventional CBT programs and leverage the advantages that bCBT programs promise, understanding the demands and requirements of an integrated bCBT program is essential. Accordingly, this study aimed to discover patients’ and therapists’ attitudes and expectations from digital mental health apps to develop better-integrated bCBT protocols. Existing knowledge on how to design an efficient digital mental health app for a bCBT protocol is heavily based on qualitative data collected during pilot studies of newly developed apps [30,40,41]. Unlike those studies, this research aimed to collect input from patients and therapists who were naive in bCBT programs, which means that they were not currently receiving a bCBT treatment and were not introduced to any bCBT app for the goals of the research. Principally, we also assumed that they had no previous experience with a bCBT program as integration of digital health apps in the psychological health care system is new in Germany and there were no bCBT options at the time of data collection or earlier [42,43]. As a result, we were able to identify the needs and expectations of patients and therapists from a perspective of inexperience with bCBT programs. Such an approach would also allow us to discover if patients and therapists have different user needs in the digital working spaces within a mental health app. Developers of new apps in this field would thereby have a general perspective on the needs of users rather than user reflections guided (and directly influenced) by previous interactions with already extant platforms.

We designed this explorative study to this end. The following research questions were addressed: (1) What are therapists’ and patients’ attitudes toward integrating digital interventions into conventional face-to-face CBT? What are the expected benefits? What are their concerns? (2) Do patients and therapists have different needs or attitudes regarding digital apps? (3) How do these attitudes and needs (including differences) translate into features of a digital mental health or software app?

## Methods

### Setting

We used a qualitative research design for this study. Qualitative methods are especially suitable for studies based on explorative research as these methods enable deriving direct insights from this study without presuppositions from earlier research [44,45]. We conducted semistructured interviews with both CBT-oriented psychotherapists and patients who were in psychotherapeutic treatment at the time of the study to unravel the perspectives of both sides of a therapeutic relationship. We deliberately targeted a patient sample with depression as the advantages of bCBT over face-to-face therapy appear most evident in this patient population [25,29,33,34]. Patients and therapists were given an explanatory introduction to the concept of bCBT programs to ensure a common understanding of the target topic of inquiry before the interviews took place. In these interactions, we made clear that our focus was on simultaneous bCBT protocols that present digital apps within the same period as face-to-face treatment [18]. We communicated that our goal would ultimately be to develop a digital app based on and incorporating the participants’ feedback.

### Participants and Recruitment

#### Patients

We conducted one-to-one interviews with 11 patients who were at the time of the interviews undergoing an individual standard face-to-face psychotherapy program (ie, no group therapy) for the treatment of unipolar depression at practices associated with the training institute Academy of Behavioral Therapy in Cologne, Germany. In total, 82% (9/11) female patients and 18% (2/11) male patients (mean age 33.27, SD 11.78; range 22-57 years) participated in the study. We approached patients via email based on referrals from participating therapists from the same training institute. Inclusion criteria for patients were being between the ages of 18 and 65 years, having been diagnosed with mild to moderate depression by their therapists and assessed as not currently suicidal, having sufficient German language skills, and owning a smartphone with the iOS or Android operating system with internet access. Patients were excluded if they were experiencing or had experienced psychotic symptoms or substance dependence in the past or were currently using a digital mental health app for the treatment of a mental health diagnosis. All participating patients gave informed consent to take part in this study and provide researchers with personal data.

#### Psychotherapists

We conducted 3 focus group interview sessions with a total of 18 therapists and 5 individual therapist interviews. Focus groups and individual interviews were combined to obtain practitioners’ collective opinions within the context of interaction with their colleagues as well as individually, where topics were addressed with greater specificity. Overall, we had 23 psychotherapist participants in our study (n=19, 83% female and n=4, 17% male; mean age 31.91, SD 8.11; range 25-58 years). All therapists participating in the study were either certified practitioners (7/8, 88% female and 1/8, 12% male; mean age 39, SD 10.38; range 28-58 years) or psychotherapists in their last year of training (12/15, 80% female and 3/15, 20% male; mean age 28.13, SD 2.33; range 25-33 years), and all were practicing CBT. Except for 1 certified therapist in the focus groups interviews who had a PhD in clinical psychology, all therapists had a Master of Science. Participating therapists were recruited from multiple practices in Germany and via the training institute Academy of Behavioral Therapy, Cologne, Germany, and referrals made by this training institute. Potential participants were approached directly through email or telephone. Participating therapists gave informed consent to take part in this study and provide researchers with personal data.

For both samples, potential participants were approached directly through email or telephone. As we recruited participants through referrals, we did not record demographic information of those who declined participation in the study or did not respond to the invitation.

### Ethics Approval

The study was approved by the Ethical Board of the University of Mannheim (EK Mannheim 38/2020). Interviews were conducted by coauthor MS, who is experienced as an interviewer and has conducted several qualitative studies in the past focused on digitalization and product development. There was no relationship between the interviewer and participants before this study. Interviews with participants were conducted between February 2021 and May 2021. Participants did not receive any monetary compensation for taking part.

### Data Collection

#### Interviews With Patients

Semistructured interviews were used to explore patients’ attitudes, expectations, and concerns regarding digital mental health apps. The interviews with patients were conducted on the web via tele- and videoconferencing and lasted an average of 35 (SD 9) minutes. The interviews were audio recorded with the consent of the patients and transcribed within 24 hours of the completion of the interviews.

The following questions were the main points addressed and directed to patients: (1) Can you imagine a digital mental health app offering assistance to your ongoing psychotherapy? How would such an option help? Which functions would be helpful for you? How much time would you dedicate to working with such an app? (2) Which functions can you imagine within a digital mental health app? Which would you consider unhelpful or disturbing? (3) What would you think about having a digital mental health app recommended to you by your therapist? Would you feel forced to use it? (4) What would be your attitude toward sharing your therapy-related data with your therapist through the app? What would be your attitude if the app used your data to offer a better version of the program? (5) Do you have any concerns regarding receiving treatment using a bCBT program? What are they? Do you have concerns regarding data security?

#### Focus Groups and Subsequent Interviews With Psychotherapists

We first conducted focus groups to explore psychotherapists’ attitudes, expectations, and concerns regarding digital mental health apps. During the focus groups, web-based and digital tools (ie, Miro web-based whiteboard) were used to document the discussion and results, and meeting notes were taken. Focus group interviews were conducted on the web via videoconferencing, moderated by MS, and lasted approximately 3 hours on average. An assistant was present to take notes and introduce the web-based tool (ie, Miro). The focus groups were not audio recorded (notes were taken instead). The following questions guided the focus group interviews: (1) What do you expect from a digital health app? What are the pros and cons? (2) What does a day in the life of a psychotherapist look like? How can a digital health app support you? (3) How are the usual treatments of depression designed? What is special about these patients? How can a digital health app support usual treatment for depression?

Subsequent individual therapist interviews were conducted to explore specific issues that came up during the focus group sessions in more detail. Individual interviews were conducted on the web via tele- and videoconferencing and lasted an average of 39 (SD 3) minutes. The interviews were audio recorded with the consent of the psychotherapists and transcribed within 24 hours of the completion of the interviews.

### Data Analysis

The transcripts and meeting notes from the interviews were coded according to grounded theory, a systematic data analysis methodology that focuses on inductively developing abstract theoretical conceptions from empirical data [46,47]. For the coding of transcriptions, Dedoose software (version 8.0.35; SocioCultural Research Consultants) for qualitative research was used. Patient and therapist interviews were coded separately and each interview independently. First, patient interviews were coded. Then, therapist interviews were coded. As a result of qualitative analysis of patient interviews, 4 main categories (expected features of bCBT apps, expected qualities of bCBT apps, qualities to avoid in bCBT apps, and concerns regarding bCBT apps) and several subcategories within these main categories were formed by inductive category formation based on the content of the interviews and codes assigned to text passages within them. Similarly, based on therapist interviews, 5 main categories (day of a therapist, attitudes toward digital apps, expected features of bCBT apps, expected qualities of bCBT apps, and concerns regarding app integration into therapy) and several subcategories within these main categories were formed inductively. We iterated between the developing model and the data until a viable set of first-order codes, second-order themes, and aggregate dimensions was identified, stopping when we reached “theoretical saturation” [48]. A joint agreement was reached among all authors to stop recruitment when saturation was achieved. The coding procedure was conducted by multiple researchers to ensure intercoder reliability—EA completed the first round of coding the data, and MS reviewed the coding scheme. A second coding round was then performed by EA and MS. Subsequently, the coding scheme was reviewed again by EA, MS, and JAH, and some adaptations were discussed. Finally, JAH approved the categories. As there were only minor adaptations that emerged from cross-checking, 2 coding rounds were considered sufficient. The completed COREQ (Consolidated Criteria for Reporting Qualitative Research) [49] checklist can be found in Multimedia Appendix 1. The interview quotes included in this paper were translated by a professional translator from German into English.

## Results

### Patient Interviews

#### Overview

During the interviews, patients shared their conception of a digital app intended to deliver an integrated bCBT program. In general, patients had very positive attitudes toward bCBT programs. They made specific references to expected features and qualities they would like to see in the apps and those they would like to avoid. Participants also addressed their concerns regarding bCBT (ie, under which therapeutic circumstances or conditions they would not feel comfortable using a digital app). The categories, themes, and dimensions emerged from the coding of patient interviews are illustrated in Table 1.

**Table 1:** Results of the interviews with patients.

First-order categories	Second-order themes	Third-order aggregate dimensions
Keep track of details and timing of bad days Reminders on incomplete mood and symptom entries Overview of mood and symptoms through time	Mood and symptom tracking	Expected features of bCBT^a^ apps
Communication, calendar, and appointment management Easier management of questionnaires and diagnostics Help structure patient everyday activities Provide a timeline of activity history (for therapist and patient)	Organizational functions	Expected features of bCBT apps
Provide psychoeducation Engaging with therapy activities Collection of emergency therapeutic tools	Therapeutic content and resources	Expected features of bCBT apps
Need to regard the app as useful, relevant, and integrated Pleasant and easy to understand Option to use it from a computer Different media to convey content (visual, text, and auditory)	Usability	Expected qualities of bCBT apps
Personalized reminder schedule for different users Flexible tools to express oneself	Individualized features	Expected qualities of bCBT apps
Feeling of success through use Marking certain activities as “done” and seeing personal history in the form of completion steps Setting notifications when required or by choice	Gamification	Expected qualities of bCBT apps
Engagement with the app should not replace a face-to-face session Introduce app only with accompanying therapist supervision App should not encourage frequent and spontaneous contact with the therapist App should not create a potential for dependency	Priority and balance with face-to-face treatment	Expected qualities of bCBT apps
Dispersed use throughout the day is preferred Need to be able to work on the app in a quiet and personal space A total weekly amount of 1-2 hours of app use is foreseen	Use time fitted to patients’ needs	Expected qualities of bCBT apps
People with disabilities and who speak minority languages should be able to use the app Therapy app helps destigmatize psychotherapy	Inclusion	Expected qualities of bCBT apps
Mood and symptom tracking helps more efficient and realistic communication of one’s current situation to the therapist in sessions Open-ended thought record can help patients come to the sessions better equipped (specific triggers and questions recorded in a timely fashion)	Transfer to in-person sessions	Expected qualities of bCBT apps
Educating language and lifestyle tips Heavy numerical demonstration of one’s progress Advertisements and user cookies	Feeling distant and technical complexity	Qualities to avoid in bCBT apps
Too much content Overwhelming sound, color, or text Too many push notifications Excessive focus on one’s depressive thoughts and experiences	Input bombardment	Qualities to avoid in bCBT apps
Presentation of therapy activities as “must-do” Constantly aiming to advance and be happy	Pressuring patients	Qualities to avoid in bCBT apps
Recommendation by therapist is acceptable Alternative description to “prescription” wording should be used as it is too much for some patients Therapist prescription is positive if therapist explains how and why the app helps	Framing of a bCBT plan	Concerns regarding bCBT apps
App’s use of data to provide better service is acceptable Sharing relevant therapy data with therapist is meaningful and acceptable Data security is not a major theme of concern	Sharing of therapy-related data	Concerns regarding bCBT apps

^a^bCBT: blended cognitive behavioral therapy.

#### Expected Features of bCBT Apps

##### Mood and Symptom Tracking

A common desire of patients was a function through which they could keep regular track of their mood and symptoms regarding their mental health issues. In addition to recording details such as specific times when moods would occur, interviews also revealed a common need for the option to review one’s mood and symptom history whenever they either wanted or needed to look back on their treatment path:

Sometimes, for me, depressive episodes have been so often that I forget that I was doing fine two weeks ago. Getting reminded that there have been times or there are things which can make me believe I can cope with this would be helpful.

Patient 4

Patients noted that, to achieve regular mood and symptom tracking, the digital app could ask questions that are delivered to their smartphone regularly via push notifications. Some patients also noted that push notifications could indicate incomplete entries, thus serving as an additional reminder mechanism to increase program adherence. In addition to tracking their mood, patients also indicated a desire to record more detailed notes and how they coped with the situation. Hence, we would infer that adding such notes provides a richer and qualitative character to the mood-recording function.

##### Organizational Functions

Patients identified numerous possible app functions, such as a calendar, appointment management, communication with the therapist via a messaging platform, and practical help in dealing with questionnaires used for diagnostics:

Adding functions such as making appointments, canceling appointments, and rescheduling appointments would be helpful. And this also touches on the topic of achieving goals, in the sense of, “Where do I actually stand now in my therapy?” and “How many sessions does my health insurance still cover?”

Patient 8

Interviewees envisioned a digital app that offered additional help to organize their daily life, such as a personal checklist of activities and options to schedule (and receive reminders for) selected activities:

If it (app) says you’ve had a glass of water, check; you’ve brushed your teeth today, check. You’ve managed to walk 10 minutes today, check. Then those would be things for me which are somehow feasible, which help me in a depressive phase to take care of myself, but which don’t put pressure on me that I have to do them.

Patient 3

Apart from assistance in organizing one’s daily life, an app could also broadly support a patient’s psychotherapy progress by recording important tasks and activities when they were completed and providing an accurate timeline that could be reviewed:

It would be cool if one has something like a book, I mean of course digital, where it (the app) says, yes, you’ve done this or that. Where you can kind of enter milestones or something, because I don’t even remember a lot of things.

Patient 2

A patient highlighted that such a timeline would benefit not only patients but also their therapists in overviewing patient history. In this way, functions within the app (such as a digital journal) could offer another channel of communication between therapists and patients that takes place outside of sessions.

##### Therapeutic Content and Resources

Patients expressed their willingness to use a digital app to maintain the connection with their therapist via therapy-related material outside of regularly scheduled meetings:

I would find that perfect if it (the app) would provide clarification about how psychotherapy works and which specific form of therapy is the one that I have been working out with my therapist.

Patient 8

A common point addressed by several patients concerned “emergency” situations—specific instances in daily life where the patients felt a notable sudden deterioration in their feelings, symptoms, or thought patterns. Patients drew attention to the difficulty of remembering possible intervention techniques to make themselves feel better in such moments. A collection of a toolkit for harder moments offered through the digital app was seen as a very useful support function during such episodes:

When you’re suddenly in a situation where you’d like to see the therapist right away but she’s not there...having some tools in the app that can calm you down and keep yourself under control for that moment [would be helpful].

Patient 10

As a result, app developers should consider including options where access to such interventions is at the topmost layer such that patients can easily access such information as needed in a crisis.

#### Expected Qualities of bCBT Apps

##### Usability

Patients expressed a common need to see the digital app as relevant to their treatment goals and easy to integrate into their daily routine:

I would use it frequently if I can somehow integrate it into my everyday life. If I have the need to look into it in the morning and if it becomes part of my everyday life, like part of my routine, then I would use it frequently.

Patient 6

Patients would like an app that is easy to understand and looks esthetically pleasing. The app should contain various kinds of media (text, pictures, graphics, or audio) to present content to create a pleasant user experience:

I’m a purely visual person. I totally struggle when something contains only textual processing. I’m a picture person. So if it’s intuitive and presented with pictures, I find it easier to use.

Patient 5

A specific reference to the option of using the app in a desktop format was made by 18% (2/11) of the participants (because of the possibility of typing faster on a computer).

##### Individualized Features

The need for an individualized program was the most commonly raised issue in interviews among all patients. Interviewees reported that they would like to engage with a digital app that can be tailored to the needs of individual users:

It would definitely be good to be able to set it up in such a way that you can have it individualized, that it is related to your therapy progress, that you get a list of what’s coming up or what you need to do at a certain time, depending on how you feel or how receptive you are.

Patient 1

Patients also advocated for flexibility in how they expressed themselves. They recommended open-ended questions or customizable settings instead of only multiple-choice questions or standard options such as emojis to record their mood and feelings:

How many smileys would I have to choose from, six, seven, eight? What if the worst smiley was a crying smiley but I don’t cry on a bad depression day. For me a day where I cry is when I can admit to myself that things aren’t going the way I want them to but when I’m much more open about it and also communicate with people about it. That means a crying smiley would not be the definition of disaster for me.

Patient 4

##### Gamification

The need to include gamification elements in a digital app emerged in the interviews. To maintain continuous use of the app over a longer time—which is very relevant for psychotherapy—patients wanted to experience a feeling of success. Such a moment could be ticking some items on a checklist as “done,” with goals broken down into smaller components to be accomplished in a certain order. As a patient put it, “I’m just super gamified in my consumption, and I also just jump at reward systems...I think it’s very pleasant in the design when you see that you’ve done something when you complete something” (Patient 3).

##### Priority and Balance With Face-to-face Treatment

Every patient expressed the need to see the digital app as well integrated with face-to-face psychotherapy treatment. They highlighted that good integration and engagement with the app should never lead to replacing the face-to-face sessions with a therapist. They were unanimous that such an app should not be offered without accompanying therapist supervision:

I really think the interpersonal contact with the therapist is the key to success in the whole story...The combination of real sessions and the app is totally important because otherwise it’s basically a lifestyle product for me. It’s not a clinical aid if I had to use it all by myself. I think I just wouldn’t use it then.

Patient 5

At the same time, a design that enables frequent spontaneous contact with the therapist was seen as something to be avoided. A patient stated the following:

I think direct contact with therapists on the app is a bit too much to ask of the therapist. I would rather not do that.

Patient 7

Digital apps should be developed in a way that considers the risk to therapists regarding the loss of personal boundaries.

##### Use Time Fitted to Patient Needs

A total weekly amount of approximately 1 to 2 hours spent on a digital app was considered the right amount of time. Most of the participants (9/11, 82%) preferred a dispersed app use time throughout the day over a single continuous interaction. Those who preferred dispersed use patterns noted the possibility of taking advantage of the time spent on the bus or train. However, patients differed in their preferences in this respect, with a patient highlighting their need to work on the app in a quiet and personal space:

I couldn’t work on that in a noisy environment. I would really have to have my peace of mind somehow to be able to carry out such exercises.

Patient 9

Some patients noted the need for the app to accommodate potential variability in use patterns across time and that there should not be the expectation that patients sustain the same amount and frequency of use of the app at all times:

Especially at the beginning [of treatment], I can imagine that in the first few weeks you kind of spend significantly more time...When it later shifts to exercises and making appointments and reading, it could be less and then become more again.

Patient 8

##### Inclusion

The chance to contribute to ending the stigma regarding mental health issues also emerged as an expected benefit. Some patients considered the addition of a digital app to one’s daily life as a good opportunity to also integrate organic conversations on receiving psychotherapy treatment in discussions with their loved ones:

If such an app leads mental illness to become a bit more socially acceptable so that people can also talk about it—that there are offers of help and also modern means of communication between therapist and patient—I think that would be a good thing.

Patient 5

Another expectation with respect to inclusion concerned the importance of developing a digital app suitable for people with disabilities, such as visual or auditory impairments, as well as for people who speak different native languages:

It would be so supportive to have functions for visually and hearing-impaired people, and also linguistically. In Germany in particular, making the app available in Arabic or Turkish would be great. These things can really help to promote communication.

Patient 3

##### Transfer to In-Person Sessions

There emerged an expectation in patients that having an integrated digital mental health app should also positively translate to face-to-face sessions. Easy access to mood and symptom-tracking functions would enable patients to talk about their symptoms more realistically. Noting which events or thoughts triggered specific moods or symptoms as well as questions that occurred to them would help patients come to sessions more aware and, therefore, use the time more efficiently:

You would then (with the app) actually be able to use the therapy hours more efficiently because reviewing your timeline (in the app) would already have gotten a bit involved in your thoughts about your progress.

Patient 5

#### Qualities to Avoid in bCBT Apps

##### Feeling Distant and Technical Complexity

Patients converged on the point that they did not want to engage with a digital app that used an overly educational or distant voice with a “know-it-all” tone. Stereotypical advice such as simple lifestyle tips were identified as potential examples of this:

If you always have something appearing on your screen saying “go out in the sun” and “breathe in some fresh air” or such—I would drop something like that.

Patient 2

Content that is difficult to understand by lay audiences, the overuse of numbers, and demonstrating patient progress through the excessive use of statistics or visuals would be too demanding for patients. Even though patients acknowledge that graphics and numerically charting progress could potentially be very helpful, they expressed concern about such materials not being easy to understand and use by the audience. Patients also reported working with a program that did not display advertisements or use cookies to be desirable.

##### Input Bombardment

Most patients (7/11, 64%) expressed concerns about being overwhelmed by a digital app. Too much content; design aspects involving too much sound, color, or text; and frequent notifications sent to the patient’s phone were some examples of excessive input that they noted. Some interviewees underlined that frequent questions on mood and symptoms might lead patients to focus excessively on their depressive feelings and experiences and be drawn into a depressive spiral. This risk should also be kept in mind when deciding how frequently a digital app asks patients to reflect on their depression.

##### Pressuring Patients

Therapy activities involving the digital app should not be presented as “must” assignments (as such framing would be either harmful or counterproductive) but rather as suggestions. This approach would fit better with the patients’ vision of the digital app filling the role of a “supportive companion”:

In my case, in a burnout case, it would have been counterproductive if it (the app) had told me, “but now you have to do at least five exercises until the next session.”...If I said that voluntarily to myself, it’s okay, but if it had been associated with pressure like that, it would not be good. I would probably have dropped out.

Patient 5

Patients emphasized that it would be a substantial drawback to using a digital app if it constantly pressured them to be happy. Questions prompting patients to record their moods and overview history should not give the impression that the patient can only record good feelings as allowing for negativity and embracing it is an essential part of the therapy:

When depressed it might be nice sometimes to see, oh I’ve actually got out of bed three times now. It’s great that I get this push notification telling me that. But I believe that it could also be very brutal if things are conveyed too positively. I am not a therapist but I know that my therapist has often told me that I get up too often and don’t allow myself to say I’m sick today.

Patient 4

#### Concerns Regarding bCBT Apps

##### Framing of a bCBT Plan

In the interviews, we asked patients what they would think about having a bCBT program recommended by their psychotherapist. For most patients (6/11, 55%), such a recommendation would be viewed positively, both because of natural curiosity regarding a new therapeutic medium and trust in their therapist. A few patients (5/11, 45%) balked at the idea of their therapist “prescribing” a bCBT program, considering it unhelpful and “too much” in the context of psychotherapy:

Prescriptions always have something brutal about them. I mean, if the therapist were to say to me, “The app that we both will have is really helpful and we need to use it.” That would make it sound a thousand times better to me than saying, “I’ll prescribe it for you.” Prescription always has something very, very stringent, like it is unavoidable, in my opinion. I would have a problem with my therapist saying that I have to use this app.

Patient 8

For those who were concerned about the idea of having a digital program prescribed to them, simply having their therapist explain to them why they needed to use it and how it could help was sufficient to resolve their resistance. Patients’ doubts were instead centered on how the “prescription” was presented to them.

##### Sharing of Therapy-Related Data

The possibility of sharing data with their therapist through the app and with app developers to enhance programs were specific issues that interviewers posed explicit questions about to patients. Surprisingly, the fact that patients would have to share a lot of personal data with a digital app was not a major point of concern for them. Hence, data security concerns appeared to be an unlikely reason for the target audience to walk away from a digital app. Overall, patients were very willing to share personal data this way, with an offhand remark by a patient—“If the app can do that, why not?” (Patient 1)—being representative of the attitudes of the patients as a whole. It is noteworthy that sharing data with the app providers was an acceptable option only if their data were anonymous, secured, and not used for any other purposes.

### Therapist Interviews

#### Overview

During the interviews, therapists’ daily routines and their organization and preparation workload outside of sessions were first addressed. We explored their usual work habits to better understand the issues that may benefit from additional support from a bCBT plan. Interviewed therapists were open to and curious about digital smartphone app use in the context of psychotherapy. They identified potential beneficial app features and qualities that could enhance the quality of their workday. Participants also voiced their concerns regarding bCBT (ie, points that should be critically and carefully evaluated before deciding to work with a bCBT plan). The categories, themes, and dimensions emerged from the coding of therapist interviews are illustrated in Table 2.

**Table 2:** Results of the interviews with therapists.

First-order categories	Second-order themes	Third-order aggregate dimensions
Planning sessions and becoming familiar with patient history Note taking after the session Analog versus digital organization of therapy documents	Nature of organizational work	Day of a therapist
Appointment management is stressful Therapy worksheets get lost or are difficult to organize Insufficient current digital organization tools Paper-based documentation makes planning difficult (especially outside the office) Session planning and after-session notes are sometimes lengthy Guidebooks for planning sessions are too rigid and difficult to adapt to individual patients	Problems that emerge during the workday	Day of a therapist
Practicality Open and curious toward apps	Attitudes toward app use	Attitudes toward digital apps
Positive attitudes toward app use in psychotherapy Therapy app could potentially enhance therapeutic relationship Prescription of therapy app fosters adoption Unsatisfactory current therapy apps	Attitudes toward app use in psychotherapy	Attitudes toward digital apps
Documentation and writing session protocols Support session preparation via patient history and therapeutic resources Appointment management Monitoring of patient progress	Benefits of bCBT^a^ apps for therapists	Expected features of bCBT apps
Mood and symptom tracking Collecting therapeutic toolkit for harder moments Better questionnaire and diagnostic management Offer and remind regarding therapy resources and interventions Encourage independence of patients	Benefits of bCBT apps for patients	Expected features of bCBT apps
Ease the organizational workload and protect in-session time Contribute to the connection between patient and therapist Extra help in structuring therapy Visualization of achieved milestones and successes	General benefits of bCBT apps	Expected features of bCBT apps
Personalization of intervention content Dynamic versus monotonous experience to support changes in use over time Easy and intuitive to use No advertisements	High usability	Expected qualities of bCBT apps
App should not distort therapist work-life balance App should not replace in-person sessions but complement them App should preserve seriousness of therapy	Concerns regarding therapeutic qualities of a bCBT app	Concerns regarding app integration into therapy
Data security Wasting time with unhelpful or addictive features	General concerns regarding smartphone apps	Concerns regarding app integration into therapy

^a^bCBT: blended cognitive behavioral therapy.

#### Day of a Therapist

##### Nature of Organizational Work

The work outside of sessions with patients consists mainly of preparation for the upcoming sessions and taking notes about the session after patients leave. Therapists also have to note and manage appointment schedules and share documents with the patient’s insurance company.

For the organization and collection of therapy-related files (session protocols, questionnaire results of patients, homework papers, and insurance papers), some therapists used a paper-based file collection, whereas others used digital software that is designed to organize this work. Therapists similarly differed in their preference for use of paper-and-pen calendars versus digital systems for appointment management. Interestingly, many therapists noted that they used their existing personal calendars for this purpose and not a distinct work calendar.

##### Problems That Emerge During the Workday

A common problem for both therapists and patients was that homework sheets were difficult to manage in their paper-based format. A therapist spoke for many when she shared that “these worksheets somehow get lost or the patients don’t bring them to session. And that means they can’t do them” (Therapist 4).

A similar problem with paper-based organization systems is the case therapy documentation that therapists have to manage. Documents are typically stored in huge folders in the office such that it is difficult to manage them and they are impossible to work with outside the office:

I find it really annoying if it’s only on paper because then I can’t look at them again at home. I’m not in the office every day and sometimes I think of the patients and want to check something, but then I would have to go to the practice first to check or ask a colleague to look at the file.

Therapist 5

However, those therapists who used digital software for documentation purposes were also rarely satisfied with the programs they had been using, with a therapist describing theirs as a “mechanical and dull program and also not that easy to work with...There are tens of spaces to click and hundreds of rows in front of you” (a therapist in the focus group discussion).

Finally, therapists noted that session planning before and note taking after sessions sometimes takes longer than planned, and the nondigital tools used to facilitate them (eg, guidebooks for planning sessions) are not helpful as they are not flexible (eg, session guidebooks follow a rigid suggestion format that is difficult to adapt to individual patients).

#### Attitudes Toward Digital Apps

##### Attitudes Toward App Use

The therapists interviewed were open and curious about digital smartphone apps in general and thought that digitalization brings about new and creative practical solutions to everyday matters. In this respect, they did not view psychotherapy as an exception and saw using a bCBT program in mental health treatment as a viable option that both therapists and patients would benefit from.

##### Attitudes Toward App Use in Psychotherapy

Therapists had diverging opinions about whether a digital ingredient within the therapeutic relationship would potentially enhance the relationship between the therapist and the patient. Some therapists stated that a digital app would contribute to improved therapeutic relationships, whereas others thought that it would not. One noted that a digital app could give patients a sense of continuing care and attention from their therapists outside of sessions:

I think it’s quite nice for some patients if they think to themselves: Oh, my therapist has already looked in the app about what I’ve been doing this week. So I think that can be beneficial for their relationship.

Therapist 1

Others expressed skepticism regarding the positive impact of a digital app on a therapeutic relationship built on face-to-face interactions within the context of a therapy session:

I don’t exactly know how an app is supposed to bond me and my patient. My experience is that the relationship simply grows from face-to-face contact and from the feelings that you feel for each other, and not from a worksheet or a digital platform.

Therapist 3

Importantly, no interviewed therapist believed that a digital app would damage the therapeutic relationship.

Therapists responded positively to the idea of prescribing a bCBT program, noting that it “does more justice” to the reality of the current generation of patients. They thought that a prescription could foster adoption by patients who already invest “two to three hours a day” in screen time and would prescribe a bCBT plan if they were convinced that the digital app supported the face-to-face therapy process “not only to deal with the therapy in the session but to accelerate this everyday transfer to promote and make it easier” (Therapist 3).

None of the interviewed therapists had suggested a psychotherapy-related app to a patient before, let alone prescribed one. The apps that the therapists were familiar with were deemed not satisfactory enough to recommend their use by patients.

#### Expected Features of bCBT Apps

##### Benefits of bCBT Apps for Therapists

Therapists remarked that digital documentation of many different kinds of therapy-related material, whether therapy activities, information on patients, or session protocols, would be helpful. A therapist specifically noted that having their session protocols digitally available would allow them to “search for keywords if you know that a topic has come up before or something” (Therapist 3). Better documentation functionality would also lead to easier planning for sessions, where therapists usually need to look at their notes and session protocols from previous meetings.

Another potentially useful contribution of a digital app would be providing therapeutic information that therapists could consult when needed:

So if I am focused on a topic, then I read up in my books, or I would look again in my personal notes. I google general things, for example grief counseling or things like that. Then I write down things that I find interesting and take them with me to the session. So yeah, I would use the app if I could access that information through it.

Therapist 4

Moreover, therapists expressed the desire to see basic information about a patient and where they stand in their therapy progress in a brief and easy-to-understand visual format:

It would be really helpful to have such an overview in which relevant diagnostics and symptom history for the patient can also be seen. And their status, information on the next session and perhaps also something like, “where does the patient stand right now in the therapy?”

Therapist 2

Finally, many therapists (3/5, 60%) cited setting and canceling appointments as well as seeing their scheduled appointments in calendar form as helpful functions in an app.

##### Benefits of bCBT Apps for Patients

Within the context of CBT treatment, receiving help from a digital app by working with therapy resources and interventions would be one of the main possible benefits. Therapists stated that the app could offer intervention activities and therapeutic content as well as reminders for patients. Although it would support patients’ between-session progress, the app would also ease administrative aspects of the work therapists do:

I have the feeling that maybe the homework would be done more often [with the app] and I wouldn’t always have to print everything out. Sometimes there’s information that I don’t feel like explaining in the session; maybe depending on the app patients could read about it.

Therapist 5

Many other functions that therapists wanted were also in line with patients’ expectations. Similar to patients, therapists named mood and symptom tracking, easier management and overview of diagnostics questionnaires, and collection of a toolkit that includes specific therapeutic interventions to use in harder moments as potentially helpful for patients. The ability to recall past data was prominently cited by many therapists:

If the app saves the data, you could also look again specifically at how it was three months ago because I’ve often had the experience that patients can’t remember.

Therapist 5

An opinion shared by a few participants (2/5, 40%) was that the opportunity to engage with therapy material more intensively during a patient’s personal time through the app would encourage the independence of patients:

It could contribute to the agency of my patients, where I wouldn’t be the sole communicator of therapeutic knowledge and advice and so on. When they read some of the stuff on the app, and do homework more often than before with the app, it could give a sense of increased control and autonomy.

A therapist in the focus group discussion

##### General Benefits of bCBT Apps

Therapists thought that both they and their patients would take advantage of functions that ease the organizational workload and, therefore, preserve limited in-session time for more efficient use. Both parties would benefit from routinely available extra help to structure the therapy. Similar to patients, therapists also brought up the potential for a sense of continued togetherness between the therapist and patient and an enhanced therapeutic relationship because of a bCBT app. In addition, the potential to visualize achieved milestones and successes would make them easier to understand and aim for by patients and therapists.

##### Expected Qualities of bCBT Apps

Unsurprisingly, therapists were interested in a smooth, easy, and intuitive experience for both themselves and their patients when using the digital app. Therapists particularly noted that, as a course of treatment for many patients typically takes months, the digital app should offer a dynamic and engaging experience instead of a potentially monotonous one. The app needs to support patients as their functions change over the course of treatment through effective notification strategies. Personalization of intervention content for each patient would also lead to patients following the bCBT protocol over a longer time. The therapists stressed that the app should not incorporate advertisements (especially in the context of a prescription app).

#### Concerns Regarding App Integration Into Therapy

##### Concerns Regarding the Therapeutic Qualities of a bCBT App

Some of the therapists interviewed (2/5, 40%) were concerned that introducing a digital medium to the psychotherapy work might give the impression of appearing overly available to patients. They especially pointed to the potential ability of patients to send them messages and commented that such a function would distort their work-life balance. A therapist suggested that the app could keep a record of such queries and that the therapist could access them to “get an idea of what has preoccupied the patient” without feeling the obligation to respond.

Another major concern was defining the role of a digital app within the therapeutic relationship. A digital app should not change the frame of in-person sessions so that its role replaces a session. It should be clear to patients that the digital program has a complementary function supporting sessions with the therapist. A therapist mentioned the concern that the digital app might potentially look too game-like, which could be counterproductive for therapy purposes. Although it should offer a user-friendly interface, it should also preserve the seriousness of therapy.

##### General Concerns Regarding Smartphone Apps

Unlike patients in our interviews, all therapists were concerned with the fact that an app they would prescribe to their patients should ensure patients’ data security. The difference between the responses of patients and therapists in this respect might be tied to the responsibility that therapists feel in the context of suggesting or even prescribing the app:

I think it’s really important for the patients that I can tell them credibly it’s safe and that the data will not be misused for any other purposes—that it won’t be the case that insurance will not pay for treatment anymore depending on what they write on there or their insurance premiums will go up.

Therapist 3

Therapists highlighted that it is also crucial to ensure that, for a digital mental health app, the known danger of addiction when using digital apps (such as social media) is not present. Patients should not waste their time with unhelpful functions or (even worse) become dependent on the app in ways that contribute to the mental health issues the patient is grappling with.

## Discussion

### Principal Findings

Our study explored patients’ and psychotherapists’ attitudes toward the introduction of a digital mental health app into their ongoing psychotherapy process, including their expected features and concerns regarding bCBT protocols. We gained valuable insights into the perspectives of both potential user groups. Both patients and therapists had positive attitudes toward bCBT and digital health apps in general. The results provide not only a rich frame of reference regarding what functions are expected from an integrated digital app but also a guide on what to avoid.

We suggest that this qualitative study is an important contribution to the relevant literature as scientific inquiry into how to construct a digital app for bCBT protocols has not been extensive enough to create a solid framework [26]. Current knowledge is mostly based on interview data with patients or therapists regarding their personal experiences with existing bCBT programs combined with initial evaluations of programs that have been developed [30,40,41]. Our study alternatively offers fruitful data on patient and therapist experiences and expectancies that were not guided by any particular digital program. As patients and therapists in our study were not actively using a bCBT program and were assumed to lack experience with such programs given that these were not available earlier [43] (and no sample intervention was provided), they were not oriented toward a specific existing digital health program while voicing their views on potentially helpful and unhelpful digital program features. In conducting such an exploration without focusing on a particular digital health app, our study resonates with the work by van der Vaart et al [50], although their study used survey questions and qualitative data in parallel. A similar methodological approach to ours was used in the qualitative investigation by Cerga-Pashoja et al [51], but they conducted their exploration only with therapists. With this study, those who wish to develop integrated blended solutions for existing problems in psychotherapy can find the voices of both patients and therapists regarding potential problems and practical solutions. To our knowledge, the explicit distinction between *adjunct* and *integrated* digital app designs in bCBT programs has not been made earlier in the literature. On the basis of studies on existing digital apps calling themselves as such [29,30], we pointed out this distinction and showed in this study that an integrated bCBT design is more desirable to potential users.

In this study, patients and therapists mostly agreed on which functions would be expected in a digital app, and the differences were few but notable. Data security proved to be one of the fundamental issues that therapists needed assurance about, whereas patients were not as concerned. Conversely, patients saw potential negative connotations attached to the prescription of a digital mental health program, whereas therapists did not. These findings inform researchers and developers working in the field of digital mental health apps on how different functional aspects were viewed positively or negatively and underline how psychotherapy is experienced differently by patients and therapists.

Our findings are in line with the existing consensus in the bCBT literature on the need for customizability of digital apps to an individual patient’s needs in terms of both content and use [26,41,50]. Both therapists and patients view the rigidity and nonexclusiveness of existing digital tools as the reasons for their current limited use [52]. Our study supports these findings. Similarly, references to a desire for high usability in a digital program were made by both therapists and patients, supporting an earlier study with therapists pointing to “intuitive usability and logical structure of online platform” as a facilitator of bCBT use [41]. Attempts to develop new digital mental health programs or modify existing ones should address these concerns.

Aside from simply replicating the findings on the need for personalization, we argued that there could be 2 different interpretations of what personalization of a digital program means. According to one interpretation of the data, different patients should not be given identical psychological intervention content or feedback, which is consistent with previous research [26,41,50,53,54]. A second interpretation suggests that some patients view any restricted means for personal expression as a feature to avoid (as in the example of smiley faces or multiple-choice questions). Patients reported that these methods were inadequate to their communication needs. To our knowledge, this call for more open-ended and expressive means of recording one’s mood and symptom history as well as personal thoughts is a new interpretation of customization in the digital mental health domain.

Previous findings on the needs of patients and therapists within the context of bCBT programs agreed that digital apps should avoid overwhelming patients [40,51]. For example, the volume of homework exercises delivered through the digital app [40] and the reminders and notifications provided [51] should not be excessive. Our findings endorse these earlier studies. Similarly, our findings supported the findings of van der Vaart et al [50] that therapists are concerned about preserving boundaries and keeping in-person therapy sessions central. Our study suggests that face-to-face and web-based elements should focus on distinct goals within a bCBT protocol, with the latter concentrating on the practical aspects of the therapy (such as homework assignments, diaries, and psychoeducation), whereas process-related matters and evaluation of the patient’s condition are reserved for face-to-face meetings with the therapist [50]. Therapists had differing views on the potential effect of digital mental health apps on the therapeutic relationship between a patient and a therapist, with some suggesting that treatment through bCBT protocols may foster this relationship, whereas others expressed skepticism. Remarkably, none of the therapists thought that digital mental health apps would hinder the relationship between patient and therapist. These findings are important considering that the therapeutic relationship is known to be one of the strongest predictors of treatment success [55-57].

Receiving extra assistance in a bCBT treatment was put forth as an important potential benefit by both patients and therapists in our interviews. Given that both patients and therapists are eager to try a digital app to promote their therapy progress, digital apps appear to offer a practical solution to widespread adherence issues regarding patients completing assigned homework [10,12].

### Limitations

As our patient sample included only those who were diagnosed with depression and was small (n=11), the needs and expectations of this patient group might not be generalizable to all patients in psychotherapy. Patients with different mental health diagnoses might have different needs and express them differently than the population we studied. Moreover, our study included a selective sample of adult patients who were already undergoing CBT-oriented therapy and were seemingly motivated and held positive attitudes regarding bCBT programs. Patients who are not yet in treatment for their symptoms, such as those on waiting lists, or those who are not receiving a first-line treatment such as CBT might hold different views from those of our sample. Similarly, we cannot make claims about the potential of bCBT programs in children and teenagers with our findings as we deliberately targeted only adult patients. Further research is needed to understand if and in what conditions nonadult users can benefit from bCBT programs. For some patients, careful evaluation of their suitability for a bCBT program might be necessary. For instance, based on our finding that therapists believe it is crucial to ensure that digital app use does not lead to excessive use of digital devices and potential addiction as a result, patients who are prone to technology addiction may need higher caution before a bCBT program is introduced to them.

A limitation of our study is that we did not ask patients about their current or previous involvement with stand-alone iCBT programs or other digital apps. Differential familiarity with such programs might potentially influence user views. Furthermore, we did not record how long participating patients had been in psychotherapy. Different experiences in terms of completed therapy duration might have led to differing opinions of patients. In contrast, examining patient views independent of the duration they had spent in therapy could also be considered a strength because, as we did not set any patient duration in therapy as an inclusion criterion, our study potentially covers a higher variety of patients. Finally, the explicit distinction we made between *adjunct* and *integrated* bCBT programs was mainly based on our observations in the literature. More research is needed to support the validity of such a clear conceptual distinction.

Our therapist sample consisted of only those working with a CBT orientation and was small (n=23). It should be noted that previous research has showed that therapists who work with a CBT orientation have more positive attitudes toward the idea of using digital and blended interventions in the context of psychotherapy compared with therapists using other psychotherapy orientations [58,59]. Thus, the results of this study might not be generalizable to all psychotherapists regardless of their therapy orientations.

All patients and therapists participating in this study resided in or close to Cologne, Germany. Hence, one cannot directly generalize findings to other cultures or other regions of Germany.

Further research is needed to investigate expectations and opinions of different patient samples as well as comparisons of participant expectations of various psychotherapy approaches.

### Conclusions

Our qualitative study found that patients and therapists who had never participated in a bCBT program could imagine themselves using one. They were willing to incorporate a digital app into their current face-to-face therapy practices. Both parties in the therapy relationship were open and curious about the possibilities and opportunities that technology could bring to their treatment journey. Participants provided a detailed framework for what features should be included in digital apps, independent of any particular mental health app introduced to them before the study. Therefore, researchers and developers of new technological solutions can use our findings as an independent guide when constructing new digital apps.

## Appendix

Multimedia Appendix 1 COREQ (Consolidated Criteria for Reporting Qualitative Research) checklist.

## Abbreviations

bCBT: blended cognitive behavioral therapy
CBT: cognitive behavioral therapy
COREQ: Consolidated Criteria for Reporting Qualitative Research
iCBT: internet-based cognitive behavioral therapy
